# Prevalence and risk factors for nerve injury following shoulder dislocation

**DOI:** 10.1007/s12306-022-00769-4

**Published:** 2022-11-29

**Authors:** C. M. Hardie, R. Jordan, O. Forker, A. Fort-Schaale, R. G. Wade, J. Jones, G. Bourke

**Affiliations:** 1 Leeds Institute for Medical Research, University of Leeds, Leeds, UK; 2 Department of Plastic and Reconstructive Surgery, Leeds Teaching Hospitals Trust, Clarendon Wing, Leeds General Infimrary, Great George Street, Leeds, LS1 3EX UK; 3 Faculty of Medicine and Health Sciences, University of Leeds, Leeds, UK; 4 Department of Emergency Medicine, Leeds Teaching Hospitals Trust, Leeds, UK; 5 Department of Integrative Medical Biology, University of Umea, Umeå, Sweden

**Keywords:** Shoulder dislocation, Upper extremity, Brachial plexus, Brachial plexus neuropathies

## Abstract

**Background:**

The glenohumeral joint dislocation can be associated with major nerve injury. The reported prevalence and risk factors for major nerve injury are variable and this injury can have a severe and life-long impact on the patient. The objectives of this study were to analyse the prevalence of major nerve injury following shoulder dislocation and examine risk factors. Management and outcomes of nerve injury were explored.

**Methods:**

A 1 year retrospective cohort study of 243 consecutive adults who presented with a shoulder dislocation was performed. Data were collected on patient demographics, timings of investigations, treatment, follow-up, and nerve injury prevalence and management. The primary outcome measure was prevalence of nerve injury. Risk factors for this were analysed using appropriate tests with Stata SE15.1.

**Results:**

Of 243 patients with shoulder dislocation, 14 (6%) had neurological deficit. Primary dislocation (*p* = 0.004) and older age (*p* = 0.02) were significantly associated with major nerve injury. Sex, time to successful reduction and force of injury were not associated with major nerve injury in this cohort. Patients with nerve injury made functional recovery to varying degrees. Recurrent shoulder dislocation was common accounting for 133/243 (55%) attendances.

**Conclusions:**

Shoulder dislocation requires careful assessment and timely management in the ED. A 6% rate of nerve injury following shoulder dislocation was at the lower border of reported rates (5–55%), and primary dislocation and older age were identified as risk factors for nerve injury. We emphasise the importance of referring patients with suspected major nerve injury to specialist services.

## Introduction

The shoulder is the most frequently dislocated major joint, with an incidence of 40.4 per 100,000 and 15.5 per 100,000 in men and women respectively [1]. There is a bimodal peak incidence, with the majority occurring in young people aged between 15–29 years, and a second peak in elderly females [1]. Approximately 33–40% of people with shoulder dislocation will suffer recurrence [2] with severe pain and obvious joint deformity being hallmarks of presentation. Pain management and diagnosis of associated fractures and nerve injuries are critical before being able to safely perform reduction [3]. As time progresses muscle spasms can make it more challenging to reduce the shoulder joint [4]. After shoulder dislocation outcomes can be complicated by: chronic instability; recurrent dislocations; long term pain; and altered neurological function [5–8]. Nerve injuries can be permanent with long-term functional deficit, particularly in the older population [8, 9]. However, there is limited evidence of the extent of shoulder dislocations associated with neurological injuries in younger age groups.

Our primary objectives were to analyse the incidence of nerve injury following shoulder dislocation and identify risk factors for this, in patients presenting to the emergency department (ED) of a single major trauma centre. Risk factors of interest included those relating to standard of care, injury related factors and intrinsic patient factors. Our secondary objectives were to explore the management and outcomes of patients sustaining nerve injury following shoulder dislocation.

## Methods

### Study design

This was a retrospective cohort study of consecutive patients who presented to the ED with suspected shoulder dislocation, between 1st March 2016 and 28th February 2017. Local approval for the work was given, formal ethical review was not required as per our institution’s guidelines.

### Participants

The study included adults (over 18 years of age) at presentation with a shoulder dislocation that required reduction. Patients were identified using clinical coding for ‘dislocated shoulder’. Exclusion criteria included; subluxations that did not require reduction, dislocations that had relocated before arrival, incorrect coding and no case notes available.

### Data collection

All data was sourced from electronic health records (patient pathway manager plus; picture archiving and management system [PACS]). We captured the time of injury; time of arrival to ED; time of first radiograph; time of analgesia; pain score (out of 10) at baseline; time from arrival to successful reduction; force of injury and neurovascular status of affected limb. Successful reduction time was taken as the documented time of reduction attempt if there was a post-reduction radiograph demonstrating a relocated shoulder. If time of reduction attempt was not documented the time of successful post-reduction radiograph was used.

Force of injury was defined as either high energy or low energy based upon our department major trauma triage guidance. High energy mechanisms were defined as: pedestrian versus car incidents; all falls from greater than 1 m or 5 stairs (including found at bottom of stairs); high speed road traffic accidents > 60 miles per hour; ejections from vehicle; all vehicle roll-overs; death in same vehicle; all road traffic accidents involving cyclists or motorcyclists; all incidents involving horses; multiple areas of injury with concerns that the patient has polytrauma. All other mechanisms were defined as low energy. A successful first reduction (yes/no) was defined as an anatomically reduced shoulder on radiograph after one documented reduction attempt. The assessment of neurovascular status in the department would comprise of evaluation of the sensory/motor function and the perfusion of the upper limb.

### Statistical methods

Data were analysed using StataSE v15.1 [10]. Wilcoxon rank sum was used to evaluate the association of age with dislocation frequency (1st dislocation vs. recurrent dislocation). The difference in age in years between the groups was calculated using Hodges-Lehmann median differences. The Chi squared test was used to evaluate the association of sex with dislocation frequency. Spearman’s correlation coefficient was used to evaluate the association of time to pain relief with time to successful reduction. Wilcoxon rank sum was used to assess the association of the variables age and time from arrival to reduction and with major nerve injury. Fisher’s exact test was used to evaluate the association of sex, dislocation frequency and force of injury with major nerve injury. Univariable logistic regression (bootstrapped using lossless non-parametric resampling with replacement, with 1000 iterations) was used to estimate the odds ratio (OR) for major nerve injury, according to age and whether the dislocation was first-time dislocation or recurrent. Confidence intervals (CI) were generated to the 95% level.

## Results

### Participants

There were 357 patients in the initial list coded with a diagnosis of a dislocated shoulder. Of these patients 243 met the inclusion criteria. Reasons for exclusion were: subluxation only (*n* = 90), incorrect coding (*n* = 17), inaccessible data (*n* = 6). The demographic data are shown in Table 1.

**Table 1:** Demographics and shoulder dislocation descriptors

Factor	*n* (%)
*Age (years) (n* = *243)*	
median (inter-quartile range)	37 (24–62)
*Sex (n* = *202)*	
Female	86 (35)
Male	157 (65)
*Affected side (n* = *243)*	
Right	148 (61)
Left	88 (36)
Bilateral	7 (3)
*Mechanism of injury (n* = *243)*	
Low energy	203 (84)
High energy	40 (16)
*Type of dislocation (n* = *243)*	
Anterior	234 (96)
Posterior	8 (3)
Inferior	1 (< 1)
*Dislocation frequency (n* = *243)*	
First dislocation	110 (45)
Recurrent dislocation	133 (55)

Patients experiencing shoulder dislocation had a median age of 37 years (males median = 34, females median = 51). The incidence was higher in males and on the right. One patient had 3 episodes of bilateral dislocations due to seizures. Low-energy injuries were more common in this cohort. For patients presenting with recurrent dislocation 64 attended once over the year, 10 attended twice, one attended three times, two attended four times, one attended five times and one patient attended 19 times. The patient attending 19 times had poorly controlled seizures which caused them to dislocate their shoulders.

Patients with recurrent dislocation were younger (median age 29 years, inter-quartile range 24–43) compared to primary dislocations (median age 57 years, inter-quartile range 34–74), *p* < 0.001. Those with recurrent dislocation were on average 18 years younger. Low-energy injury was more frequent with recurrent dislocation 118/133 (89%) compared to primary dislocation 85/110 (77%, *p* = 0.02).

Following removal of multiple attendances (40 attendances dropped) patients with recurrent dislocation were still significantly younger (median age 32 years, inter-quartile range 23–49) compared to primary dislocation (median age 57 years, inter-quartile range 34–74), *p* =  < 0.001. Patients with recurrent dislocation were on average 15 years younger. There was no association between the force of the injury and recurrent dislocations (primary dislocation 85/110 (77%) low energy versus 78/93 (84%) recurrent dislocations, *p* = 0.24).

### Prevalence and outcomes of major nerve injury

218/243 (90%) patients were assessed for neurovascular status. 14/218 patients (6%) had a neurological deficit, these patients are detailed in Table 2. Seven had global infra-clavicular brachial plexus injury, two had an axillary nerve deficit, one an axillary nerve plus suprascapular nerve deficit, two an ulnar nerve deficit, one a sensory radial nerve deficit and one a posterior and medial cord deficit. Seven patients were referred from the ED to a major nerve outpatient clinic and two patients were referred from fracture clinic. Five patients were referred to and managed by fracture clinic alone. Within this nerve injury group, one patient had brachial plexus exploration and he was managed with specialist and local therapy. One patient did not attend any follow up and one moved away. The remaining 11 patients had non-operative management with therapy. The median time to discharge from clinic was 34 weeks (range 9–97). At time of discharge patients had made functional recovery to varying degrees. Two made full recovery (sensory deficits) at discharge (both managed by fracture clinic alone), one had partial recovery of sensory deficit, four had fully recovered sensory deficits and partly recovered motor deficits, and four were discharged with some remaining mixed deficits. One patient only attended one follow up appointment with fracture clinic where they were noted to have axillary nerve numbness but did not attend any further appointments so end recovery is unknown, one patient moved abroad shortly after their injury and one attended no follow up.

**Table 2:** Demographics, injury, follow up and recovery of patients with major nerve injury following shoulder dislocation

Patient	Age	Sex	Nerve injury	Follow up clinic	Time to discharge (weeks)	Motor recovery	Sensory recovery
1	67	Female	Global plexus	Fracture major nerve	32	Full	Partial
2	77	Female	Global plexus	Fracture	36	Partial	Partial
3	47	Male	Global plexus	Fracture major nerve	16	Full	Partial
4	74	Male	Global plexus	Fracture major nerve	90	Partial	Partial
5	54	Male	Global plexus	Fracture major nerve	72	Partial	Partial
6	40	Male	Global plexus	Fracture major nerve	N/A	Unknown (DNA*)	Unknown (DNA)
7	33	Male	Sensory axillary	Fracture	11	N/A	Full
8	56	Male	Sensory axillary	Fracture	18	N/A	Unknown (lost F/U**)
9	49	Female	Sensory and motor axillary, and motor suprascapular	Fracture major nerve	42	Full	Partial
10	53	Female	Sensory ulnar	Fracture	9	N/A	Full
11	45	Female	Global plexus	Major nerve	10	Full	Partial
12	41	Female	Sensory and motor ulnar	Fracture	Unknown moved abroad	N/A	N/A
13	69	Female	Sensory radial nerve	Fracture	39	N/A	Partial
14	83	Male	Posterior and medial cord sensory and motor	Fracture major nerve	97	Partial	Partial

*DNA Did not attend, **F/U Follow up

### Risk factors for major nerve injury

Risk factors for major nerve injury were examined (Table 3). Older age was significantly associated with major nerve injury (*p* = 0.02) whereby the odds of sustaining a major nerve injury increased by 20% with each decade of life (OR 1.02 [95% CI 1.002–1.04], *p* = 0.03). Patient sex, and time from arrival to reduction were not significantly associated with major nerve injury. 9/14 (64%) patients with major nerve injury had their dislocation reduced within 2 hours. Two patients waited over 2 hours to have their dislocated shoulder reduced and three patients waited over 3 hours. The odds of sustaining a major nerve injury were sixfold higher in patients who sustained their first dislocation (OR 5.94 [95% CI 1.50–23.46] *p* = 0.01), compared to those with recurrent dislocations. Force of injury was not associated with major nerve injury, and there was a slightly higher rate compared to those without (21% of patients with nerve injury had high energy injury, compared to 16% not). Following removal of multiple attendances (35 attendances dropped) older age was still significantly associated with major nerve injury (*p* = 0.05), as was first time dislocation (*p* = 0.02).

**Table 3:** Risk factors for major nerve injury

Variable	Major nerve injury	No major nerve injury	*p*
Age, years *n* = 218 Median (range)	54 (45–69)	36 (24–62)	0.02
*Sex n* = *218*			
Female *n* (%)	7 (50)	74 (36)	
Male *n* (%)	7 (50)	130 (64)	0.54
Time from arrival to successful reduction, mins Median (range) *n* = 200	102 (49–131)	103 (70–169)	0.92
*Dislocation frequency n* = *218*			
First dislocation *n* (%)	12 (86)	88 (43)	
Recurrent dislocation n (%)	2 (14)	116 (57)	0.004
*Force of injury n* = *218*			
Low energy	11 (79)	171 (84)	
High energy	3 (21)	33 (16)	0.71

### Provision of care

115/140 (82%) patients describing moderate or severe pain (pain score 4–10/10) were offered analgesia within 30 min of arrival and 134/140 (96%) had been offered by 60 min. Time to successful reduction was not associated with time to pain relief (*p* = 0.70). 169 patients (70%) had a shoulder radiograph within 60 min of arrival. 141 patients (60%) had a first reduction attempted within 2 hours of arrival, and 192 (80%) had first attempted reduction within 3 hours. The median time from arrival in the ED to successful reduction was 97 min (range 15–406). 225 patients (93%) had a post-reduction radiograph performed. Four patients self-discharged prior to post-reduction radiograph but it is not clear why the fourteen others did not have this performed. In total 233 patients (96%) were booked for follow up: 197 (85%) in fracture clinic, 14 (6%) in other orthopaedic clinics e.g. already under upper limb surgeons, 8 (3%) with both fracture clinic and major nerve clinic follow up, and 15 (6%) had other arrangements. Three patients self-discharged.

## Discussion

The demographics of this retrospective cohort are similar to published literature in presenting a majority of younger, male patients generally with lower energy injury mechanisms [2]. However, the proportion of attendances (55%) with recurrent dislocation was high, with one patient with epilepsy accounting for 19 attendances. This high proportion of recurrent dislocation highlights the burden for patients and health services of ongoing problems with shoulder instability. Younger patients have been identified as having increased risk of dislocation, with 39% of those under 40 reporting dislocation recurrence in systematic review [11], and a reported 90% recurrence in young, athletic patients [12]. This was also demonstrated in our cohort with patients presenting with recurrent dislocation being significantly younger. There is limited evidence regarding the best management of primary dislocations to prevent recurrent instability [13]. In addition, it seems prudent that best management of co-existing conditions such as epilepsy is optimised.

Shoulder dislocations are known to cause acute, severe pain [3] and timely administration of analgesia and sedative drugs can reduce pain and muscle spasms increasing the chance of a successful reduction [14]. This relationship was not demonstrated in this study as time to successful reduction was not associated with time to pain relief administration. In this cohort 82% of patients were offered analgesia within 30 min and 96% within 60 min, demonstrating good patient care. 70% of patients received an radiograph within 60 min of arrival, which could be contributing to delays in reduction attempts which can be associated with lower reduction success and increased risk of neurological impairment [15] [16]. The time to the first reduction attempt was slow in some cases: 60% in under 2 hours and 80% within 3 hours. Potential barriers to fast reduction include other more urgent clinical priorities and availability of skilled staff, which may reflect general resource shortages within the National Health Service [17].

Documentation of a neurovascular examination of the affected limb was also not comprehensive (10% of attendances had no documentation of any neurovascular assessment). This has clinical and medicolegal consequences [18] as neurovascular deficits may be missed, resulting in poor patient care. 6% of those examined had a neurological deficit which is within previously reported rates which range from 5–40% [6–8]. The axillary nerve was the most commonly affected single nerve (2/14 patients) as is typically reported in the literature [7]. However, in seven patients a global infra-clavicular brachial plexus deficit was identified. A higher energy mechanism of injury was considered as a possible explanation but this was the case in only 2/7 patients. The high rates of global palsy may be due to the relatively small numbers of patients in this study. In addition, the hospital is a major trauma centre so ambulances will preferentially bring serious trauma to this centre.

Of the 14 patients suspected of having neurological deficit following dislocation, nine were referred to a specialist major nerve injury clinic. Although the majority of patients were managed nonoperatively, input from the specialist therapy team is recommended [19]. We found recovery to be mixed, with two patients demonstrating complete resolution but the others with persistent functional deficits. This is consistent with other work reported by our group on the recovery of patients after major nerve injury [19].

Older age was identified as a significant risk factor, as has been demonstrated by other authors [15]. The literature reports that duration of dislocation is associated with increased risk of complications [3, 4, 8] however this was not demonstrated in this cohort. This may be related to the small number of patients with nerve injury (6%). The other significant risk factor for major nerve injury identified was first time dislocation in comparison to recurrent dislocation. There is limited literature comparing rates of nerve injury between primary and recurrent shoulder dislocation. As the primary dislocation is typically of greater force than subsequent dislocations it is consistent that concomitant nerve injury is more likely at this time. It may be that the numbers in this study are too small to demonstrate a significant association between high force of injury and risk of nerve injury.

In terms of management of nerve injury following shoulder dislocation we advise that all suspected nerve injuries are referred to a specialist major nerve service, ideally from the emergency department. Low energy injuries can usually be managed conservatively in the first instance with physiotherapy, but may require nerve conduction studies if adequate recovery is not demonstrated at three weeks. If required, nerve exploration and reconstruction can be undertaken within 3 months and no later than 6 months. High energy injuries carry a higher suspicion of serious nerve injury hence should have a lower threshold for early nerve exploration (days to weeks).

The limitations of this study were that it was retrospective with some incomplete data and the number of patients sustaining major nerve injury is small. There were some bilateral cases and repeat attendances meaning that there is dependency in our data which has not been accounted for. The time taken for the first reduction attempt was variable and delayed in some cases, which may affect the applicability of this study. Please contact authors if data is required.

## Conclusion

Shoulder dislocation requires immediate assessment and timely management in the ED. Nerve injury was identified in 6% of patients presenting with shoulder dislocation which is consistent with current literature. We also report the associations between older age and primary dislocation with major nerve injury following shoulder dislocation. The importance of performing and documenting a comprehensive neurovascular assessment for shoulder dislocation due to the risk of neurological injury is emphasised, as well as the need to refer patients with nerve injury to specialist services.
